# C4-Substituted Isoquinolines: Synthesis and Cytotoxic Action

**DOI:** 10.2174/1874104500701010001

**Published:** 2007-07-19

**Authors:** A Tsotinis, S Zouroudis, D Moreau, C Roussakis

**Affiliations:** 1 Department of Pharmaceutical Chemistry, Faculty of Pharmacy, University of Athens, Panepistimioupoli-Zografou, 157 71 Athens, Greece; 2 ISOMer (Institut des substances et organisms de la mer), Laboratoire de Pharmacologie Marine, Faculté de Pharmacie, Nantes, France

## Abstract

A facile synthesis of the C4-substituted isoquinolines **5a-c** and **6a-c** is described. Commercially available 4-bromoisoquinoline is converted to the *α,β*-unsaturated esters **8** and **10** on treatment with the appropriate acrylate ester under Heck reaction conditions. The saturated amides **5a-c** were obtained from the reaction of ester **9** with the requisite primary amine. Similarly the unsaturated analogues **6a-c** were prepared by reacting ester **10** with the appropriate amine. The cytotoxicity of the target molecules was evaluated in two tumour cell lines *in vitro*. Two compounds, **6b** and **6c**, showed sufficient activity in the human non-small cell lung cancer line NSCLC-N16-L16 to be worthy of further study.

## INTRODUCTION

**Fig. (1):**
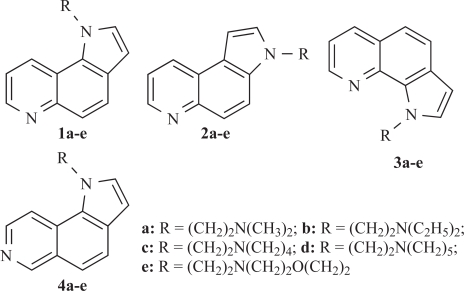
Structures of derivatives of 1*H*-pyrrolo[2,3-*f*]quinoline **1a-e**, 3*H*-pyrrolo[2,3-*f*]quinoline **2a-e**, 1*H*-pyrrolo[3,2-*h*]quinoline **3a-e** and 1*H*-pyrrolo[2,3-*f*]isoquinoline **4a-e**.

Suitably substituted quinolines and isoquinolines constitute the principal subunits of a large number of linear and angular aromatic “frameworks” with cytotoxic properties (Langer Clin Cancer Res 1999) [1] (von Nussbaum JMC 1999) [2]. These molecules interact either with topoisomerase II or bind in the major or minor groove of DNA. In some cases they form DNA-intercalated molecular complexes, which are stabilized *via* hydrophobic interactions, hydrogen bonding and/or van der Waal’s forces (Morrell JMC 2006) [3]. The presence of nitrogen bearing side chain(s) in specific positions of their skeletons improves binding affinity and enhances solubility under physiological conditions (Werbel JMC 1986) [4]. Previously, we have published the synthesis of the cytotoxic pyrroloquinoline derivatives **1-3** (Fig. 1) (Vlachou Heterocycles 2002) [5] and their isomeric pyrroloisoquinolines **4a-e** (Fig. 1) (Vlachou EJPS 2002) [6].

**Scheme 1.**
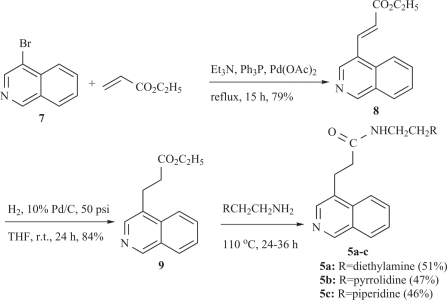
Synthesis of the new saturated amides **5a-c**.

**Scheme 2.**
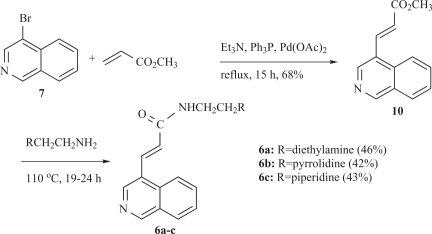
Synthesis of the new *α,β*-unsaturated amides **6a-c**.

In these molecules the spatial orientation of the side chain with respect to the planar aromatic chromophore appears to influence their cytotoxic potency and DNA-binding affinity. Based on these findings we present herein the synthesis and biological activity of the C4-substituted isoquinolines **5a-c** (Scheme 1) and **6a-c** (Scheme 2), which constitute structurally related congeners of **1-4**. For the synthetic plan we had to start with commercially available 4-bromoisoquinoline (**7**), which was reacted with ethyl acrylate under Heck reaction conditions (Sakamoto Heterocycles 1981) [7] to give *α,β*-unsaturated ethyl ester **8** in 79% yield as a single isomer of *trans* geometry. Catalytic hydrogenation of **8** over Pd/C in THF under 50 psi pressure led to the formation of the saturated ethyl ester **9**, which was converted to the desired amides **5a-c**, upon heating (110^o^C) with the appropriate primary amine [8].

However, analogous treatment of the unsaturated ester **8** with the same amines did not lead to the desired compounds **6a-c** in a satisfactory yield. The situation was rectified by substituting ethyl acrylate **8** with its methyl congener **10**, which was prepared from 4-bromoisoquinoline (**7**) and methyl acrylate under Heck conditions (Scheme 2). The new 4-isoquinoline propanamides **5a-c** and their unsaturated counterparts **6a-c** were examined for *in vitro* cytotoxicity in a panel of two tumour cell lines (Table 1). In the leukemia K562 cell line none of the tested compounds showed any noticeable potency. Although most of the compounds tested were also inactive in the human non-small lung cancer cell line NSCLC-N16-L16 (an IC_50_ of 10 μM or lower is generally considered as “active” in anticancer drug discovery), the unsaturated analogues **6b** and **6c**, with IC_50_ values 44.0 and 35.6 μM, respectively, are considered as active in this particular tumour and suitable candidates for further biological evaluation.

It is noteworthy that few agents, e.g. dactinomycin, are clinically effective against human non-small lung cancer, and thus there is a need for novel agents for use in this disease.

**Fig. (2):**
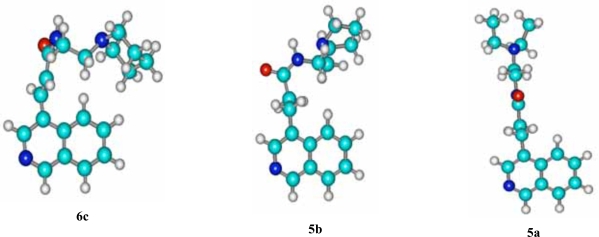
Energetically favoured conformations of **6c**, **5b** and **5a**; the structures were obtained with the aid of HyperChem™ 7.0 Molecular Modeling System.

The fact that the unsaturated piperidino analogue **6c** is marginally more active in this line than its congener **6b** can be possibly attributed to the increased basicity of piperidine (pyrrolidine’s pk_a_=11.11; pk_a_ of piperidine =11.29 [9]). The lack of potency in NSCLC-N16-L16 shown by the saturated analogues **5a** and **5c** is probably due to the different spatial orientation of their flexible side chains. Conversely, it seems that the “locked” geometry of the side chains of **6b** and **6c** allows them to interact in a more favorable way with the backbone of DNA (Tsotinis LDDD 2005) [11] (*vide* 3D structure of **6c** against **5a**, Fig. 2). It is interesting that the spatial disposition of the side chain of the weak saturated non-small lung cancer growth inhibitor **5b** resembles that of **6c** is (Fig. 2).

## CONCLUSIONS

A short synthetic route to C4-substituted isoquinolines is described. Two of the analogues produced, **6b** and **6c**, have cytotoxicity in the NSCLC-N16-L16 cell line and may provide new leads in the search for effective cytotoxic agents.

## Figures and Tables

**Table 1:** Cytotoxicity of the New Analogues in the K562 and NSCLC-N16-L16 Carcinoma Cell Lines

Compound	K562 (IC_50_ in μM)*a*	NSCLC-N16-L16 (IC_50_ in μM)*b*
**5a**	> 100	Inactive
**5b**	> 100	124.4 ± 7.6
**5c**	Inactive	Inactive
**6a**	> 100	Inactive
**6b**	> 100	44.0 ± 1.2
**6c**	Inactive	35.6 ± 0.9

a IC_50_ values were measured by using a microculture tetrazolium assay (3-(4,5-dimethylthiazol-2-yl)-2,5-diphenyl-2*H*-tetrazolium bromide, MTT) following a 72 h exposure to drug at 37°C.

b IC_50_ values were determined by using the MTT assay; three concentrations of the drugs were tested in duplicate and cell growth was evaluated at 72 h (Mosmann J Immunol Methods 1983) [10].

## References

[1] Langer SW, Schmidt G, Sørensen M, Sehested M, Jensen PB (1999). Inhibitors of topoisomerase II as pH-dependent modulators of etoposide-mediated cytotoxicity. Clin Cancer Res.

[2] von Nussbaum F, Miller B, Wild S (1999). Synthesis of 1-(2-aminophenyl)iso-quinolines and the biological activity of their cis-dichloro platinum(II) complexes. J Med Chem.

[3] Morrell A, Antony S, Kohlhagen G, Pommier Y, Cushman M (2006). A systematic study of nitrated indenoisoquinolines reveals a potent topoisomerase I inhibitor. J Med Chem.

[4] Werbel LM, Angelo M, Frei DW, Worth DF (1986). Basically substituted ellipticine analogs as potential antitumor agents. J Med Chem.

[5] Vlachou M, Tsotinis A, Kelland LR, Thurston DE (2002). A new ring-forming methodology for the synthesis of bioactive pyrroloquinoline derivatives. Heterocycles.

[6] Vlachou M, Tsotinis A, Kelland LR, Thurston DE (2002). An expeditious synthesis of cytotoxic pyrroloisoquinoline derivatives: Structure–activity comparative studies with isomeric pyrroloquinolines. Eur J Pharm Sci.

[7] Sakamoto T, Arakida H, Edo K, Yamanaka H (1981). Synthesis of pyrimidine derivatives having olefinic substituents by palladium-catalyzed cross-coupling reaction of iodopyrimidines. Heterocycles.

[R8] 8Satisfactory analytical data/NMR spectra were obtained for all new compounds; this set of data is available free of charge as supplementary material upon request. Typical experimental procedure: **Synthesis of *N*-(1-piperidinethyl)-4-isoquinolinepropenamide (6c)**. A mixture consisting of 4-bromoisoquinoline (**7**) (2.10 g, 10.1 mmol), methyl acrylate (1.12 g, 13.0 mmol), triethylamine (1.7 mL), triphenylphosphine (0.06 g, 0.23 mmol) and palladium(II) acetate (0.03 g, 0.13 mmol) was refluxed under argon for 15 h. Upon cooling the resulting suspension was treated with water (5 mL) and ethyl acetate (20 mL) and filtered through celite. The filtrate was extracted ethyl acetate (3 x 25 mL), and the combined organic phases washed with brine. After drying over Na_2_SO_4_, the solvent was removed *in vacuo* to leave an oily residue, which was purified by flash column chromatography (eluent: cyclohexane/ethyl acetate, 95:5) to give 0.75 g (68%) of 4-isoquinolinepropenoic methyl ester (**10**) as an off-white solid. Mp 75-76°C (ethanol). ^1^H-NMR (400 MHz, CDCl_3_): δ 3.78 (s, 3H, OCH_3_), 6.58 (d, 1H, CHCOO, *J*=15.7 Hz), 7.60-7.68 (m, 1H, H_7_), 7.73-7.81 (m, 1H, H_6_), 7.99 (d, 1H, H_8(5)_, *J*=8.2 Hz), 8.11 (d, 1H, H_5(8)_, *J*=8.2 Hz), 8.34 (d, 1H, CH=CHCOO, *J*=15.7 Hz), 8.72 (s, 1H, H_3_), 9.22 (s, 1H, H_1_). ^13^C-NMR (100 MHz, CDCl_3_): δ 51.7, 121.6, 122.3, 127.4, 127.9, 128.1, 131.0, 133.4, 138.7, 141.4, 153.8, 166.5A mixture consisting of ester **10** (0.21 g, 0.98 mmol) and 1-(2-aminethyl)piperidine (0.25 g, 1.95 mmol) was stirred at 110°C for 19 h. Upon cooling the excess of amine was removed *in vacuo* and the residue formed was treated with water (5 mL) and ethyl acetate (20 mL). The aqueous layer was separated and extracted with ethyl acetate (3 x 25 mL). The combined organics were washed with brine and dried (Na_2_SO_4_). The solvent was evaporated under reduced pressure to leave a residue, which was triturated with cyclo-hexane to give 0.13 g (43%) of the desired amide **6c** as an off-beige solid. ^1^H-NMR (400 MHz, CDCl_3_): δ 1.39-1.45 (m, 2H, N(CH_2_CH_2_)_2_CH_2_ piperidino), 1.52-1.60 (m, 4H, N(CH_2_CH_2_)_2_CH_2_ piperidino), 2.34-2.42 (m, 4H, N(CH_2_CH_2_)_2_CH_2_ piperidino), 2.49 (t, 2H, CH_2_N(CH_2_CH_2_)_2_CH_2_, *J*=6.2 Hz), 3.49 (q, 2H, NHCH_2_, *J*=6.2 Hz), 6.56 (d, 1H, CHCOO, *J*=15.3 Hz), 6.63 (bs, 1H, NH), 7.58-7.63 (m, 1H, H7), 7.70-7.75 (m, 1H, H_6_), 7.95 (d, 1H, H_8(5)_, *J*=8.3 Hz), 8.13 (d, 1H, H_5(8)_, *J*=8.3 Hz), 8.28 (d, 1H, CH=CHCOO, *J*=15.3 Hz), 8.69 (s, 1H, H_3_), 9.17 (s, 1H, H_1_). ^13^C-NMR (100 MHz, CDCl_3_): δ 24.3, 25.8, 36.2, 54.3, 57.1, 122.9, 125.1, 127.6, 128.2, 131.0, 134.9, 141.1, 153.4, 165.2. Anal Calcd for C_19_H_23_N_3_O (%): C, 73.76; H, 7.49; N, 13.58; found: C, 73.55; H, 7.38; N, 13.41

[9] (1989). The Merck Index.

[10] Mosmann T (1983). Rapid colorimetric assay for cellular growth and survival: Application to proliferation and cytotoxicity assays. J Immunol Methods.

[11] Tsotinis A, Vlachou M, Zouroudis S (2005). A facile synthesis of C2-substituted pyrrolo[2,3-*f*]quinolines with cytotoxic activity. Lett Drug Des Discov.

